# 5-Hydroxymethylcytosine (5hmC) at or near cancer mutation hot spots as potential targets for early cancer detection

**DOI:** 10.1186/s13104-022-06028-w

**Published:** 2022-04-21

**Authors:** Michael J. Lu, Yabin Lu

**Affiliations:** Anchor Molecular Inc., New York, USA

**Keywords:** 5hmC, Cancer hotspots, DNA methylation

## Abstract

**Objective:**

Universal noninvasive genomic screening to detect cancer and/or fetal DNA in plasma at all stages of development is highly warranted. Since 5-hydroxymethylcytosine (5hmC) emerged as an intermediate metabolite in active DNA demethylation, there have been increasing efforts to elucidate its function as a stable modification of the genome. In the current study, we demonstrate that discrete 5hmC sites within 80 bp hotspot regions exist in a greater proportion of cancer versus normal cells.

**Result:**

5hmC was detected in 16 of 17 known hotspots having C to T or G to A mutations. The results show the presence of two characteristically distinct 5hmC groups: Tier 1 Group with 3 to eightfold more 5hmCs detected in tumor-cells than in normal-cell derived DNA (as observed in 6 of 11 CpG sites). Tier 2 group with equal allele frequency of 5hmC among normal and tumor-cell derived DNA at 5 CpG hotspot sites as well as 5 non-CpG hotspots. Thus, detection and quantification of the Tier 1 group of 5hmC sites or its prevalence at or near cancer mutation hot spots in cells may enable early detection, screening and potentially prediction of the likelihood of cancer occurrence or the severity of the cancer.

## Introduction

Noninvasive methods for detection of cancer or fetal DNA in plasma include DNA methylation. DNA methylation, at the 5-position of cytosine to form 5-methylcytosine (5-mC), which occurs predominantly in the context of CpG dinucleotides, is one of the best-characterized epigenetic modifications in processes [1, 2].

In 2009, two reports in *Science* indicated that mammalian genomic DNA contains not only 5-mC but also 5-hydroxymethylation (5hmC), which is now widely regarded as the “sixth base” in the mammalian genome [3, 4]. In one well-known mechanism, 5hmC is produced from 5mC in an enzymatic pathway involving three 5mC oxidases, Ten-eleven translocation (TET)1, TET2, and TET3. Formation of 5hmC from 5mC lowers the levels of 5mC genome wide. It was speculated that the conversion of 5mC into 5hmC could be the first step or precursor to a pathway leading towards DNA demethylation [5]. However, the biological role of 5hmC remains unclear.

To date, no studies have examined 5hmC directly at base-resolutions within any gene coding region, especially regions surrounding known mutation hotspots known to play active roles in cancer development. In this study, we use chemical oxidation and reduction technique combined with Next Generation Sequencing (NGS) to investigate the novel existence of 5hmC on CpG sites located at and around mutation hotspots. We demonstrate an association of increased quantity of 5hmC within hotspots in cancer/tumor-derived cells but not normal cells. These findings suggest 5hmC as a novel marker and potential precursor to mutation at cytosine and thus means for distinguishing abnormal cancer cells from normal cells.

## Main text

### Materials and methods

#### Source of DNA

In the first study, normal cell DNA is extracted from one patient’s peripheral blood mononuclear cells (PBMCs) purchased from iXCells Biotechnologies. Two cell lines were used to represent tumor cells, PAM3005 and HCT116. PAM3005 cells are fibroblasts transformed by SV40 T antigen. It harbors both BRCA1 p.P871L and BRCA2 p.N372H heterozygous mutations. HCT116 cells are colon cancer derived and obtained as a gift from Max-Planck-Innovation GmbH. In the second study, genomic DNAs extracted from three pairs of matched colon cancer and normal tissue were purchased from Biochain Institute.

#### Genomic DNA preparation in the first study

Extraction and gDNA digestion were performed following the method by Brand M, et al. [6]. Frozen cells were thawed on ice before washing twice with PBS followed by two washes with Buffer N. The cells were resuspended in Lysis Buffer containing 0.3% NP40. Lysed cells were centrifuged at 524×*g* in a tabletop centrifuge for 5 min to collect the nuclei. Nuclei were resuspended with Buffer N. After determining the gDNA concentration, MNase was added to the suspension at 1.25 mU per µg of DNA for 10 min incubation at 37 ºC. The reaction was stopped with MNase Stop Buffer. The fragmented gDNA was treated with Proteinase K followed by purification with Phenyl:Chloroform extraction and ethanol precipitation.

#### 5hmC oxidation

We followed the 5hmC oxidation procedure described by Liu et al. [7]. Briefly, 5hmC DNA oligonucleotide (46 μl) was denatured with 2.5 μl of 1 M NaOH for 30 min at 37 °C in a shaking incubator, then oxidized with 1.5 μl of solution containing 50 mM NaOH and 15 mM potassium perruthenate (KRuO4, Sigma-Aldrich) for 1 h on ice. The product was purified using Micro Bio-Spin 6 Columns following the manufacturer’s instructions.

#### Pyridine Borane reduction

As described by Liu et al. [7], oxidized DNA (50–100 ng) in 35 µl of water was reduced in a 50 µl reaction containing 600 mM sodium acetate solution (pH 4.3) and 1 M pyridine borane for 16 h at 37 °C and 850 rpm in an Eppendorf ThermoMixer. The product was purified using Zymo-Spin columns.

##### NGS

Genomic DNA (gDNA) was treated with KruO4 followed by Borane to convert the 5hmC to uracil (T). An amplicon-based NGS panel was designed to sequence 50 variant targets, 33 of which are single nucleotide variant (SNV). In the first study, we examined 17 of the 33 SNV targets with 80 bp regions centered around either C or G base at each hotspot. In the second study, 80 bp regions of all 33 SNV were examined.

### Results

#### Identification of two groups of 5hmC at Hotspots

In the first study, we examined the presence of 5hmC at 17 hotspots and their flanking regions (around 80 bp with hotspot at center). The 17 hotspots were the only C- or G- containing targets in our NGS panel targeting 33 most popular SNVs. 11 of the 17 hotspots have CpG sites located right at the hotspot location. Genomic DNA (gDNA) was treated with KruO4 followed by Borane to convert the 5hmC to uracil (T). The 80 bp regions of each hotspot were sequenced by NGS. The differences in allele frequency (AF) of 5hmC between cfDNA fragments from normal and cancer cells were compared.

Allele frequencies of 5hmC at each hotspot are shown in Table 1. DNAs from normal cells (PBMC) and the two cancerous/tumor cells were compared. Both base C and G of the CpG were examined. Those AFs higher than 8% were highlighted as red and those between 4 and 8% were highlighted as orange. In cancerous cells, most CpG hotspot sites have both the C and G in the CpG island mutated.

**Table 1:** Detailed AF% of detected 5hmc at 17 Hotspots (Top: Tier 1; Bottom: Tier 2)

Chromosome	Tier 1 Hotspot Location	Mutation name	Hotspot Mutation	Bases at hotspot	C of CpG Normal (PBMC)	G of CpG Normal (PBMC)	C of CpG Immprtalized (PAM3005)	G of CpG Immprtalized (PAM3005)	C of CpG Cancer (HCT116)	G of CpG Cancer (HCT116)
chr11	108247071	ATM R337C, c.1009C>T	C>T	CpG			2.1%	***56.8%***	0.7%	***8.1%***
chr19	11021837	SMARCA4 T910M, c.2729C>T	C>T	CpG		2.0%	*6.1%*	1.1%	2.2%	***10.7%***
chr2	208248388	IDH1 R132H, c.395G>A	C>T	CpG		1.4%	2.0%	***9.1%***	2.8%	0.5%
chr1	162776212	DDR2 R709, c.2125C>T	C>T	CpG	*7.1%*	0.3%	***8.9%***	***9.6%***	*7.4%*	***20.5%***
chr17	39711955	ERBB2 S310F, c.929C>T	C>T	CpG	3.8%		***12.5%***		***16.6%***	
chr2	211623993	ERBB4 R711C, c.2131C>T	G>A	CpG	1.3%	2.1%	***8.5%***	***25.3%***	***9.9%***	*7.8%*
chr12	25245350	KRAS G12C, c.34G>T	C>A	CC			***22.58%***		***32.14%***	

Chromosome	Tier 2 Hotspot Location	Mutation name	Hotspot Mutation	Bases at hotspot	C of CpG Normal (PBMC)	G of CpG Normal (PBMC)	C of CpG Immprtalized (PAM3005)	G of CpG Immprtalized (PAM3005)	C of CpG Cancer (HCT116)	G of CpG Cancer (HCT116)
chr17	7673802	TP53 R273H, c.818G>A	C>T	CpG	1.2%	*4.1%*	2.5%	*4.8%*	*5.0%*	3.3%
chr17	7674220	TP53 R248Q, c.743G>A	C>T	CpG	*5.8%*	0.6%	***8.7%***	0.4%	*5.5%*	2.4%
chr7	55181378	EGFR T790M, c.2369C>T	C>T	CpG	*7.4%*	*4.8%*	6.4%		*6.0%*	0.7%
chr17	7675088	TP53 R175H, c.524G>A	C>T	CpG	2.3%	*4.8%*	0.8%	*6.3%*	3.4%	6.6%
chr5	112839941	APC R1450, c.4348C>T	G>A	CpG		*6.3%*		*6.7%*		***18.6%***
chr15	66436824	MAP2K1 P124S, c.370C>T	C>T	CC	*7.2%*		*5.7%*		3.8%	
chr14	104780214	AKT1 E17K, c.49G>A	C>T	CC	2.8%		1.9%		*4.7%*	
chr3	41224646	CTNNB1 S45F, c.134C>T	C>T	TCT	2.1%		1.5%		1.0%	
chr3	179218303	PIK3CA E545K, c.1633G>A	G>A	TGA		2.6%		*4.2%*		*5.4%*
chrX	71119404	MED12 G44D, c.131G>A	G>A	GG		*5.1%*		*5.4%*		*5.9%*

**Fig. 1:**
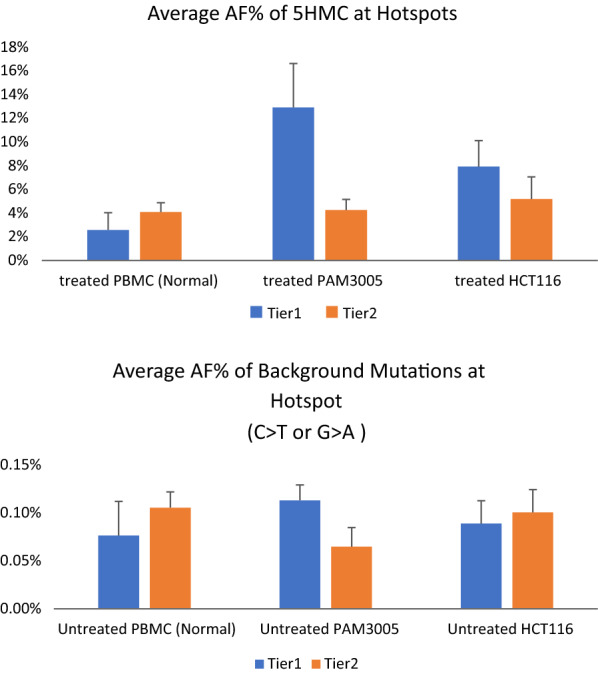
Average Allele Frequency (AF%) of detected C>T (or G>A) at hotspots before and after treatment

Two groups with similar characteristics were observed: (1) Tier 1 (top half of Table 1): with significantly more 5hmCs detected in cancerous cell gDNA than in normal gDNA (as seen in 6 of the 11 CpG sites and 1 CC site (KRAS G12C); (2) Tier 2 (bottom half of Table 1): with roughly equal allele frequency of 5hmC detected among normal and cancerous cell DNA in 5 CpG hotspot sites and 5 non-CpG hotspots. Averaged AF for each group, before or after the 5hmC-to-T conversion, were plotted in Fig. 1. Significantly higher 5hmC AF were observed in both PAM3005 and HCT116 in Tier 1, while the AF were comparable among Tier 2. Background level of C > T/G > A for all groups was comparable.

### Normal and cancer cells have similar number of 5hmC, most of which are shared with comparable allele frequencies (Tier 2 5hmC sites)

Not just at hotspots, 5hmCs were detected in about a quarter of the total CpG sites within the seventeen 80 bp regions centered around genomic hotspots. There are a total of 726 C or G associated with the CpG sites around the 17 hotspots in the first study. The number of 5hmC detected were 171, 191 and 185 for the normal PBMS cell, the immortalized PAM3005 and the colon cancer tumor cell HCT116, respectively. 158 of the 5hmC sites were shared by all 3 cell types. Most of the 5hmC sites are Tier 2 which showed comparable AF between gDNA from cancerous and normal cells (Data not shown). Similarly, in the second study using matched tumor-versus-normal DNA, the average number 5hmC sites was 1208 for tumor and 1181 for normal, when AF was above 1%.

### Cancerous cells have significantly higher 5hmC at Tier 1 sites at or near hotspots (Tier 1 5hmC sites)

**Fig. 2:**
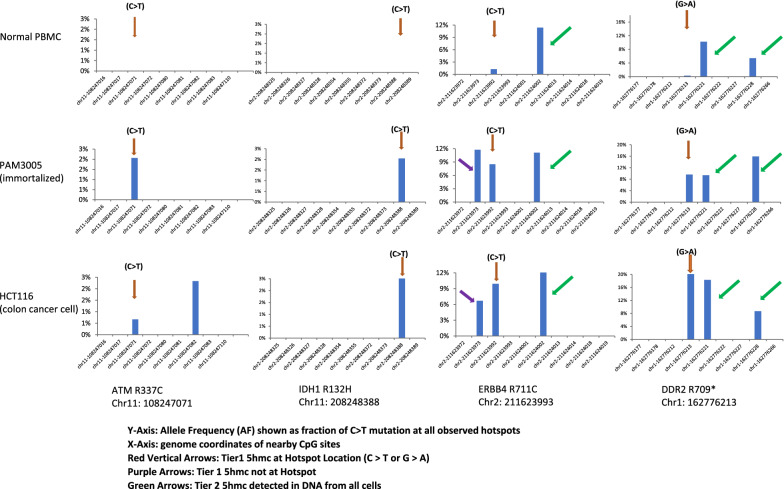
Tier 1 and Tier 2 Group 5hmcs At or Near Hotspots

Figure 2 shows a few typical Tier 1 and Tier 2 5hmC. Most of the CpG sites are “5hmC-free”. Tier 1 5hmC are shown both at hotspot (red) or near hotspot (purple). Green arrows indicate the Tier 2 5hmC.

Out of the 726 CpG sites in the first study, we identified 47 Tier 1 sites where cancerous cells exhibited significantly higher AF of 5hmC. Only 3 sites were found to have significantly higher 5hmC in normal cell than from cancerous cells.

In the second study, more 5hmC sites were observed in tumor than normal DNA at higher AF. For example, at 5% AF or above, an average of 606 5hmC sites were found in each tumor DNA versus 442 in normal DNA. At 10% or higher, the average number was about 76 in tumor versus 36 in normal. The number of extra 5hmC (Tier 1) found in tumor was proportionally higher in high AF range. Calculated as percentage of 5hmC sites found in normal, there were 2%, 37%, 110%, and 229% more 5hmC counts in tumor, and 22%, 45%, 124% and 255% higher sum of AF values in tumor than in normal gDNA, when detection criteria of AF were set at above 1%, 5%, 10 and 15%, respectively.

The significantly increased 5hmC AF in cancerous/tumor cells implies the potential role of 5hmC in cancer formation and its utility as a cancer marker.

### Discussion

Efforts in using mutation hotspots as cancer biomarkers have not been fully successful since cancer is usually associated with many mutations. Even the most popular hotspots often do not show up in most cancer cases. Universal markers like methylated cytosine (5-methylcytosine or 5mC) and Tumor Mutation Burden (TMB) have been widely explored as simple markers [8, 9]. However, both markers still lack large-scale validation, precluding implementation in clinical practice [10, 11].

5hmC is a rather common modification of cytosine among all cell types. Majority (80–92%) of these 5hmC are shared, with comparable AF, by both normal and cancerous cells, except the Tier 1 group. The association of increased allele frequency of Tier 1 5hmC to hotspots and its vicinity CpG sites in cancer or transformed cells potentially provides another simple way to distinguish cancer cells from normal cells. Because 5hmC is not detected by normal sequencing techniques as mutated, the increased detection of certain 5hmC groups at hotspots is likely a more sensitive marker of cancerous cells occurring before many mutations (e.g., C to T changes) happened.

Studies on the functional role of 5hmC have been heavily focused on change in chromosome-wide global 5hmC density or concentration, or regulation of transcription in the promoter region, or loss of 5hmC across many types of cancer [12, 13]. Unlike the uniform distribution of 5mC outside of the promoter regions, satellites, and repeat DNA sequences [14], 5hmC has distinct distributions across different functional regions, and its abundance varies across different tissues and cell types [15–17]. Tissue type plays a dominant role in determining the distribution patterns of 5hmC [18]. 5hmC is enriched primarily in the distal regulatory regions, gene bodies of actively expressing genes and promoters, indicating its connection with active transcription [19]. Genome-wide analysis of 5mC has indicated the global hypo-methylation pattern in tumor tissues, whereas depletion of 5hmC has also been associated with the hyper-methylation of gene bodies in various cancers [18, 20, 21]. Significant enrichment of 5hmC is observed in both tissue-specific and cancer-specific differentially methylated regions as compared with that of 5mC [22]. Other discoveries include the findings that several genes coding for proteins involved in the 5mC oxidation reaction are mutated in human tumors and that there is a broad loss of 5hmC across many types of cancer. The disruption of TET2 and IDH1/2 gene function by mutations perturbs 5-hmC levels in hematopoietic stem and progenitor cells and has been shown to participate in the pathogenesis of hematopoietic malignancies. Conflicting results have been reported on inhibition of TET and suppressed hydroxymethylation (5hmC), such as promoting somatic cell reprogramming [23], increased gene expression of tumor suppression [24], and reduced cholangiocarcinoma progression [25].

Moreover, the significant increase of Tier 1 group of 5hmC at many hotspots in cancer cell lines may suggest a previously unknown higher order mechanism underlying the development of hotspot mutation and cancer. 5hmC is a base that is modifiable into uracil (T). Coincidently, many hotspots with the CpG site (6 of the 11 in our case) frequent have both the Cs on both strands mutated to T. It is interesting to investigate the potential role of 5hmC in the formation of C > T hotspot mutations, because, if this hypothesis is true, markers along the 5hmC-mediated mechanism or pathway in cancer development are not only better diagnostic targets than mutations at hotspots, but also potentially better therapeutic targets. Drugs directly or indirectly either prevent 5hmC from occurring, prevent 5hmC from being converted to uracil- or thymine-analog, or correct 5hmC back to regular cytosine may prevent or treat cancer. Our results further support the need for studies to validate the clinical role of 5hmC levels in differentiating normal from tumor cells.

## Limitations

This work was limited by the number of cells in both the normal and the cancerous cells groups. Further study with increasing number of samples, larger number of hotspots and wider regions flanking the hotspots are needed.

## Data Availability

Materials described in the manuscript, including all relevant raw data, will be freely available to any scientist wishing to use them for non-commercial purposes, without breaching participant confidentiality. Please contact Dr. Yabin Lu for any data request.

## Abbreviations

5hmC: 5-Hydroxymethylation
AF: Allele frequency
5-mC: 5-Methylcytosine
NGS: Next Generation Sequencing
